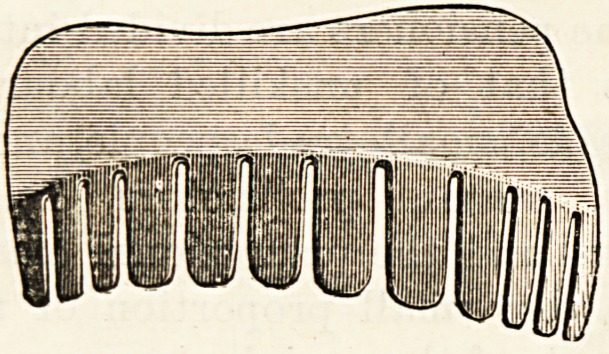# New Appliances and Things Medical

**Published:** 1899-05-13

**Authors:** 


### NEW APPLIANCES AND THINGS MEDICAL.

[We shall be glad to receive, at our Office, 28 & 29, Southampton Street, Strand, London, W.C., from the manufacturers, specimens of all new preparations and appliances which may be brought out from time to time.]

### NEW INSTEP ARCH.

(London Shoe Co., 123, Queen Victoria Street and 116 and 117, New Bond Street.)

This is a device which has been patented by Dr. Davies for the support of the instep in cases of flat foot or where the sufferer has to remain standing for many hours at a time. The discomfort which arises from flat foot or weakness of the ligaments which support the instep arch is well known to nurses, shop assistants, and waiters, and many inventions have been brought out from time to time to correct the tendency. Dr. Davies' patent consists of a thin piece of steel, solid for one half, and cut into a series of tooth-like springs on the other half. The latter give to a certain extent when pressure is brought to bear by the weight of the body, and when kept in position under the foot give a considerable degree of support to the arch of the foot. The metal piece is covered by a casing of kid, and held in position by an elastic strap. It can be worn with either boots or shoes, and appears to be an excellent corrective to flat foot or a tendency thereto.

### ELIXIR PHOSPHORI.

(O. E. Horn, Glendower, Bournemouth.)

This preparation is specially devised to solve the difficulty of dispensing free phosphorus in an agreeable form and under conditions that do not affect the therapeutic value of the element, and yet ensures the stability of its chemical constitution. One drachm of the elixir is reputed to contain 1-50th of a grain of phosphorus. It will mix with water, and offers no incompatibility when combined with iron and quinine or their preparations. As a nerve tonic a stable preparation of this kind should be of great value.

### ROBINSON'S PATENT BARLEY AND GROATS.

(Keen, Robinson, and Co., Garlick Hill, London, E.C.)

Robinson's patent barley is a specially prepared form of the grain for the rapid making of barley water. There are several ways of so doing, but perhaps the best is that given under "Advice for Mothers by a Mother" at the beginning of a small pamphlet supplied by the manufacturers. Barley water as a diluent of milk for infants is held in deservedly high repute, but a caution must here be insisted upon, namely, that the barley water must not be too strong—it is probable that half the strength usually recommended is amply sufficient. Barley water should be added not with the idea of supplying an element or elements of food, but with the object of diluting the milk with a mucilaginous liquid which renders the clotting of the casein, or curd, of a finer and less heavy character than when milk is simply diluted with water. Robinson's patent barley is eminently suited for the preparation of nursery barley water. The process to which it has been subjected appears to favour the digestibility of the decoction, and the various samples we have inspected appear of uniformly good quality. The patent groats prepared by the same firm can be conveniently employed in the making of porridge for older children; they are rapidly and easily cooked.

### ESVACH WATER.

(Davy, Hill, and Son, Yates and Hicks, 64, Park Street, Southwark.)

This water is of mild aperient qualities, and having, at tho same time a slight degree of alkalinity, it is specially indicated in cases where habitual constipation is combined with general acidity. It is bottled in screw-stoppered bottles, most convenient for travelling or occasional use. It is, however, in cases where it is systematically taken in small doses every morning, say, half a wine-glass, that its value will best be appreciated.

### S. CHARLES EVAPORATED CREAM.

(S. Charles Condensing Co., Illinois, U.S.A.)
(Agents, Cosenza and Co., 95, Wigmore Street, Cavendish Square.)

The preparation under notice is prepared from milk produced in the Elgin district. It is evaporated *in vacuo* to the consistency of thick cream, and owing to this process of sterilisation is absolutely free from living germs and bacteria. It contains no added sugar, and, consisting solely of the evaporated product of the cow, it can, by corresponding dilution with water, be again converted into milk with the natural proportion of the constituent elements. For children and invalids it is of special value, as the contained casein, owing to the prolonged sterilisation, is rendered more amenable to gastric digestion.

## Figures and Tables

**Figure f1:**